# Pulse Oximetry: A Non-Invasive, Novel Marker for the Quality of Chest Compressions in Porcine Models of Cardiac Arrest

**DOI:** 10.1371/journal.pone.0139707

**Published:** 2015-10-20

**Authors:** Jun Xu, Chen Li, Liangliang Zheng, Fei Han, Yan Li, Joseph Walline, Yangyang Fu, Dongqi Yao, Xiaocui Zhang, Hui Zhang, Huadong Zhu, Shubin Guo, Zhong Wang, Xuezhong Yu

**Affiliations:** 1 Emergency Department, Peking Union Medical College Hospital, Chinese Academy of Medical sciences, Beijing, China; 2 Emergency Department, Tianjin Medical University General Hospital, Tianjin, China; 3 Emergency Department, Beijing Hospital, Beijing, China; 4 Institute of Life Monitoring, Mindray Corporation, Shenzhen, China; 5 Division of Emergency Medicine, Department of Surgery, Saint Louis University Hospital, Saint Louis, Missouri, United States of America; 6 Emergency Department, Beijing Tsinghua Chang Gung Hospital, Beijing, China; Azienda Ospedaliero-Universitaria Careggi, ITALY

## Abstract

**Objective:**

Pulse oximetry, which noninvasively detects the blood flow of peripheral tissue, has achieved widespread clinical use. We have noticed that the better the quality of cardiopulmonary resuscitation (CPR), the better the appearance of pulse oximetry plethysmographic waveform (POP). We investigated whether the area under the curve (AUC) and/or the amplitude (Amp) of POP could be used to monitor the quality of CPR.

**Design:**

Prospective, randomized controlled study.

**Setting:**

Animal experimental center in Peking Union Medical Collage Hospital, Beijing, China.

**Subjects:**

Healthy 3-month-old male domestic swine.

**Interventions:**

34 local pigs were enrolled in this study. After 4 minutes of untreated ventricular fibrillation, animals were randomly assigned into two resuscitation groups: a “low quality” group (with a compression depth of 3cm) and a “high quality” group (with a depth of 5cm). All treatments between the two groups were identical except for the depth of chest compressions. Hemodynamic parameters [coronary perfusion pressure (CPP), partial pressure of end-tidal carbon dioxide (P_ET_CO_2_)] as well as AUC and Amp of POP were all collected and analyzed.

**Measurements and Findings:**

There were statistical differences between the “high quality” group and the “low quality” group in AUC, Amp, CPP and P_ET_CO_2_ during CPR (P<0.05). AUC, Amp and CPP were positively correlated with P_ET_CO_2_, respectively (P<0.01). There was no statistical difference between the heart rate calculated according to the POP (F_CPR_) and the frequency of mechanical CPR at the 3^rd^ minute of CPR. The *F*_*CPR*_ was lower than the frequency of mechanical CPR at the 6^th^ and the 9^th^ minute of CPR.

**Conclusions:**

Both the AUC and Amp of POP correlated well with CPP and P_ET_CO_2_ in animal models. The frequency of POP closely matched the CPR heart rate. AUC and Amp of POP might be potential noninvasive quality monitoring markers for CPR.

## Introduction

Both the American Heart Association (AHA) and the European Resuscitation Council (ERC) 2010 guidelines for cardiopulmonary resuscitation (CPR) emphasize the need for high quality CPR, including compressions to a depth of at least 5cm and a rate of at least 100 min^-1^[1, 2]. Such “high-quality” CPR is currently felt to be the best way to achieve a successful return of spontaneous circulation (ROSC), and help patients survive cardiac arrest[1]. An inexpensive, noninvasive and quantitative hemodynamic monitor that can help rescuers adjust their performance to maximize artificial circulation to patients suffering cardiac arrest is urgently needed. Quantitative waveform capnography and invasive arterial pressure monitoring are recommended for physiologic monitoring of the effectiveness of chest compressions in the 2010 guidelines[3]. However, both of these methods are expensive, invasive or depending on advanced airway, and are not available in most CPR situations[4].

Pulse oximetry, which is noninvasive and inexpensive, is widely used in most clinical situations to monitor the oxygenation of peripheral blood. Several types of probes are commonly used, such as a finger clip, nasal clip, or forehead sticker. The operating principles of which are similar – a light detector and a light source with two waves which differ in their absorbance by oxyhemoglobin and reduced hemoglobin. Most of the light is absorbed by the fixed tissue such as the skin, muscle, bone and venous blood in a constant amount. The absorbance of the arterial blood varies with each beat, and this variance is transformed into an electric signal which is then transferred from the detector as a periodical waveform with each beat, namely the pulse oximetry plethysmographic waveform (POP) that reflects the peripheral tissue perfusion[5–7]. In patients suffering cardiac arrest, no blood flow is detected in the peripheral tissue, and, as a result, no waveform can be transported (or detected).

In our clinical practice, we found that when CPR was performed, POP regularly showed with each compression, and we also noticed that the “better” (i.e. deeper) the compression was performed, the “better” (i.e. larger) the waveform seemed. Recently, more and more studies had shown that POP could be used as an early indicator of changes in circulation[5, 8, 9]. Thus we wondered if it could be feasible to seek circulatory information from the POP during CPR.

We hypothesized that POP could reliably reflect the quality of the CPR beat by beat—both the frequency of chest compressions and their hemodynamic effect. In this study, we analyzed differences in coronary perfusion pressure (CPP), partial pressure of end-tidal carbon dioxide (P_ET_CO_2_), and the area under the curve (AUC) and amplitude (Amp) of POP between two different quality CPR programs and studied the relationship between the POP values, CPP and P_ET_CO_2_ to get some information about whether these parameters could be used to assess the quality of CPR. Finally, we analyzed the relationship between the frequency of POP and chest compressions to determine whether the frequency of POP could accurately reflect the frequency of chest compressions.

## Materials and Methods

### Preparation

This experimental protocol was approved by the Animal Care and Use Committee at Peking Union Medical College Hospital. A total of 34 domestic pigs (weight, 28±2 kg) received Pentobarbital Sodium 30 mg/kg IM (Merck, 719F034, Germany) followed by inhalational 4% isoflurane (ABBOTT, H20059911, USA) via a snout mask with 100% oxygen via an anesthesia apparatus (Veterinary Anesthesia Ventilator, Midmark Corporation, USA). After endotracheal intubation and mechanical ventilation initiation, anesthesia was maintained with intravenous propofol (Corden Pharma S.P.A., H20100645, Italy) (5~10mg/min). Each pig was mechanically ventilated in volume control mode without PEEP (VT = 8~10ml/kg, Rate = 12/min) (Esprit Ventilato, V1000, Germany), and the tidal volume was adjusted to maintain P_ET_CO_2_ at approximately 35~45 mmHg. An arterial blood gas sample was analyzed to verify the baseline conditions.

Pigs were placed in the supine position and fastened in a special “U” shape frame to avoid the pigs moving during the chest compressions. Right femoral arteries were cannulated with 6 Fr single-lumen arterial catheters with their distal tips in the thoracic aorta to monitor blood pressure. Central venous catheters (18Ga, 20 cm, Arrow, USA) were inserted from the right internal jugular veins to the right atria for right atrial pressure (RAP) monitoring. Another catheter was inserted into the left external jugular vein to place an electrode catheter in order to induce VF. Electrocardiogram (ECG) and P_ET_CO_2_ (Capnostat 5, Respironics, CT, USA) were continuously monitored. A pulse oximetry sensor (Mindray Biological Medical Electronic Co, Ltd, Shenzhen, China) was fixed around the tails of the models to obtain the POP. The ECG, invasive arterial blood pressure (ABP), RAP, P_ET_CO_2_ and POP were monitored and recorded by a T8 Mindray monitor (Mindray Biological Medical Electronic Co, Ltd, Shenzhen, China).

### Protocol Description

**Fig 1:**
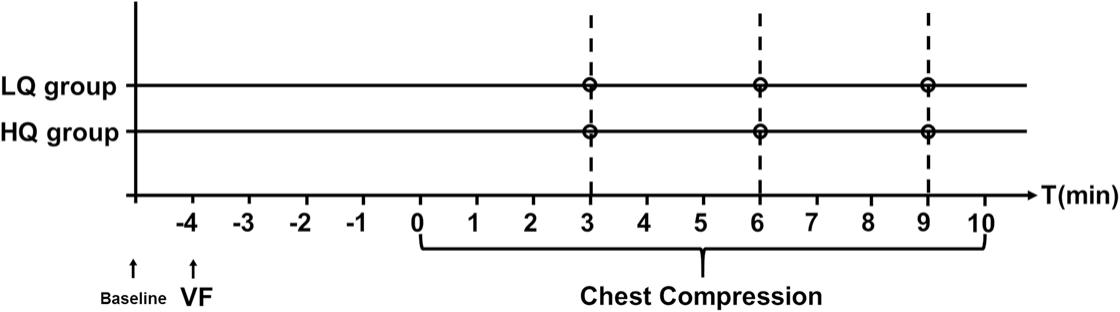
Experimental Protocol.

After baseline data was collected, a pacing electrode was positioned in the right ventricle. Ventricular fibrillation (VF) was induced by 24V/50Hz AC current applied to the right ventricular endocardium for 1 second. Once VF was confirmed by the ECG waveform and a precipitous decline in aortic pressure, the ventilator was disconnected from the endotracheal tube temporarily. After 4 minutes of untreated VF (mimicking a bystander recognizing cardiac arrest and calling for help), resuscitation was started. Once chest compressions were started, the ventilator was reconnected to the endotracheal tube and ventilated to the original parameters. All the animals were randomly allocated into two groups using random number table when grouping the animals (Fig 1): (1) a low quality resuscitation group (LQ group)–which was provided continuous chest compressions to a depth of 3cm at a rate of 105 compressions per minute; (2) a high quality resuscitation group (HQ group)–which was provided continuous chest compressions to a depth of 5cm at a rate of 105 compressions per minute. Each group had 17 pigs and received different depth chest-compressions via a mechanical CPR device (WISH-SL-FS-A, Wuhan, China). During the resuscitation, hemodynamic parameters, as well as P_ET_CO_2_ and POP were continuously monitored. 10 minutes after the end of data collection, the animals were euthanized with potassium chloride. To avoid the influence of resuscitation drugs (such as epinephrine, vasopression, etc.), no other medication was given and all treatment between the two groups was the same except for the depth of chest compressions.

### Data Acquisition

The following parameters were monitored: heart rate (HR), systolic arterial pressure (SAP), diastolic arterial pressure (DAP), mean arterial pressure (MAP), right atrial pressure (RAP) and P_ET_CO_2_. CPP during CPR was calculated by subtracting right atrial relaxation (“diastolic”) pressure from aortic relaxation (“diastolic”) pressure[10–12].

The AUC, Amp and frequency of POP were acquired by the T8 monitor and stored in Compact Flash cards. The data was analyzed by Mindray POP viewer V8.0 (Mindray Research Center for Monitoring and Life Support) using MATLAB software V7.10.0 (MathWorks, Natick, Massachusetts, USA). In order to reduce the influence of noise, the pulse oximetry signal was limited to a frequency of 11 Hz and only the near-infrared signal was analyzed. Pulse oximetry signals include the fluctuant component (*S*_*AC*_) and the constant component (*S*_*DC*_). Since the *S*_*AC*_ is related to pulsating blood volume, and the *S*_*DC*_ is related to non-pulsating tissues such as muscle and bone, we focused on the fluctuant component *S*_*AC*_ by filtering out the constant component *S*_*DC*_ in the original signals with FIR/IIR filtering technology. All the POP parameters were calculated based on the fluctuant component *S*_*AC*_.

The frequency of mechanical chest compressions was compared to the frequency of the fluctuant component (*f*_*SAC*_) waveform monitored by POP (*F*_*CPR*_).

In order to get the stable value of the amplitude, the mean square root method was used to extract the absolute amplitude value (*Amp*_*CPR*_) of each single pulse wave in the fluctuant component. The formula was as follows:

$${Amp}_{CPR}=\sqrt{\frac{\sum_{n=0}^{N-1}S_{AC}^{2}(n)}{N}}$$

A point-by-point integral method was used to calculate the absolute area parameter. The formula was as follows:

$${Area}_{CPR}=\sum_{n-0}^{N-1}S_{AC}(n)$$

[Note: *S*_*AC*_ (n) represented the n^th^ sampling data point of a single pulse wave, and N represented the total sampling point count of a single pulse wave. *Amp*_*CPR*_ represented the absolute amplitude value of a single pulse wave.]

### Statistical Analysis

Statistical analysis was performed with SPSS 16.0 for Windows (SPSS, Inc.). The normality of the distribution of each variable was assessed using the Kolmogorov-Smirnov test. The One-Sample test was used to assess the frequency variation in samples compared to the theoretical value. F-test and Student’s T-test were applied to the normal distribution data and Mann-Whitney U test was applied to the non-normal distribution data to determine differences between the two groups. *P* value < 0.05 was considered to be statistically significant.

We used the Pearson correlation coefficient for linear correlation analysis. *P* value < 0.01 was considered to be statistically significant according to Bonferroni correction.

Normal distribution data was shown as *mean*±*SD* and non-normal distribution data was shown as median (25%-75%).

## Results

In HQ group, there was an animal with extreme values. We removed that data to reduce error.

No ROSC was achieved during the initial 9 minutes of CPR. Hemodynamic parameters, P_ET_CO_2_, and the POP parameters were similar in the two groups at baseline, as shown in Table 1. However, when VF was induced, all the parameters changed not only during VF but also during chest compressions.

**Table 1:** Physiological parameters at baseline.

Group	HR (bpm)	CPP (mmHg)	P_ET_CO_2_ (mmHg)	Amp (PVA)	AUC (PVPG)
LQ group (n = 17)	95±26	102±25	40±3	477±264	4268±563
HQ group (n = 16)	96±22	102±24	39±3	445±300	4138±590

Note: Amp and AUC were calculated as the average of the data collected 30s before the detect time.

No statically significant differences were detected in any parameters between the two groups.

LQ group: low quality resuscitation group; HQ group: high quality resuscitation group; HR: heart rate; CPP: coronary perfusion pressure; P_ET_CO_2_: pressure of end-tidal carbon dioxide; AUC: area of under the curve; Amp: amplitude of POP; PVA: Pulse Oximeter Voltage Amplitude; PVPG: Pulse Oximeter Voltage Plehtysmography.

**Fig 2:**
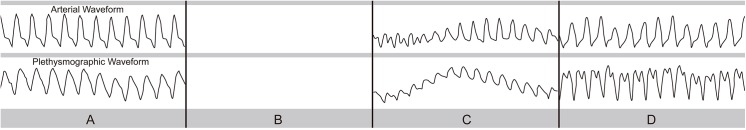
The arterial waveform and POP during different stages of CPR A. The arterial waveform and POP during spontaneous circulation at baseline; B. Both the arterial waveform and POP disappeared during VF; C. The arterial waveform and POP during low quality resuscitation; D. The arterial waveform and POP during high quality resuscitation.

When VF was induced, both the regular aortic blood pressure waveform and the POP disappeared, as shown in Fig 2. Correspondingly, the CPP, HR, AUC and Amp of POP all declined to zero suddenly. Similarly, P_ET_CO_2_ also decreased to zero after 3 minutes of untreated VF.

Not surprisingly, when resuscitation began, both the regular aortic blood pressure waveform and the POP appeared, as shown in Fig 2. All the hemodynamic parameters, P_ET_CO_2_, and the POP parameters increased significantly compared to during VF (*P*<0.05) in both groups, but all the parameters were lower than those of the baseline before VF (*P*<0.05) (Table 2).

**Table 2:** Physiological parameters during resuscitation.

		LQ group (n = 17)	HQ group (n = 16)	*t/U*	*P*
HR (bpm)	3min	104±6	104±5	0.251	0.803
	6min	103±3	103±3	0.245	0.808
	9min	103±3	103±2	0.532	0.599
	3min	12±4	19±4	-4.830	<0.001
P_ET_CO_2_ (mmHg)	6min	13±4	19±4	-3.948	<0.001
	9min	12±4	18±4	-4.690	<0.001
	3min	14.6±9.8	25.0±17.5	-2.146	0.040
	3min	14±10	21±5	-2.301	0.028
CPP (mmHg)	6min	15±10	21±5	-2.221	0.034
	9min	12±7	23±6	-4.872	<0.001
	3min	70±62	189±129	-3.317	0.003
Amp (PVA)	6min	71±63	194±132	-3.379	0.003
	9min	79±81	188±119	-3.081	0.005
	3min	2215±852	3191±556	-3.872	0.001
AUC (PVPG)	6min	2211±781	3193±517	-4.231	<0.001
	9min	2022±665	3067±522	-5.001	<0.001

Note: Amp and AUC were calculated as the average of the data collected 30s before the detect time.

PVA: Pulse Oximeter Votage Amplitude; PVPG: Pulse Oximeter Voltage Plehtysmography

There were statistically significant differences in CPP, P_ET_CO_2_, AUC and Amp between the high quality group and the low quality group at the 3^rd^, 6^th^ and 9^th^ minutes of chest compressions. When the quality of chest compressions increased, these parameters increased correspondingly (*P*<0.05) (Table 2).

**Fig 3:**
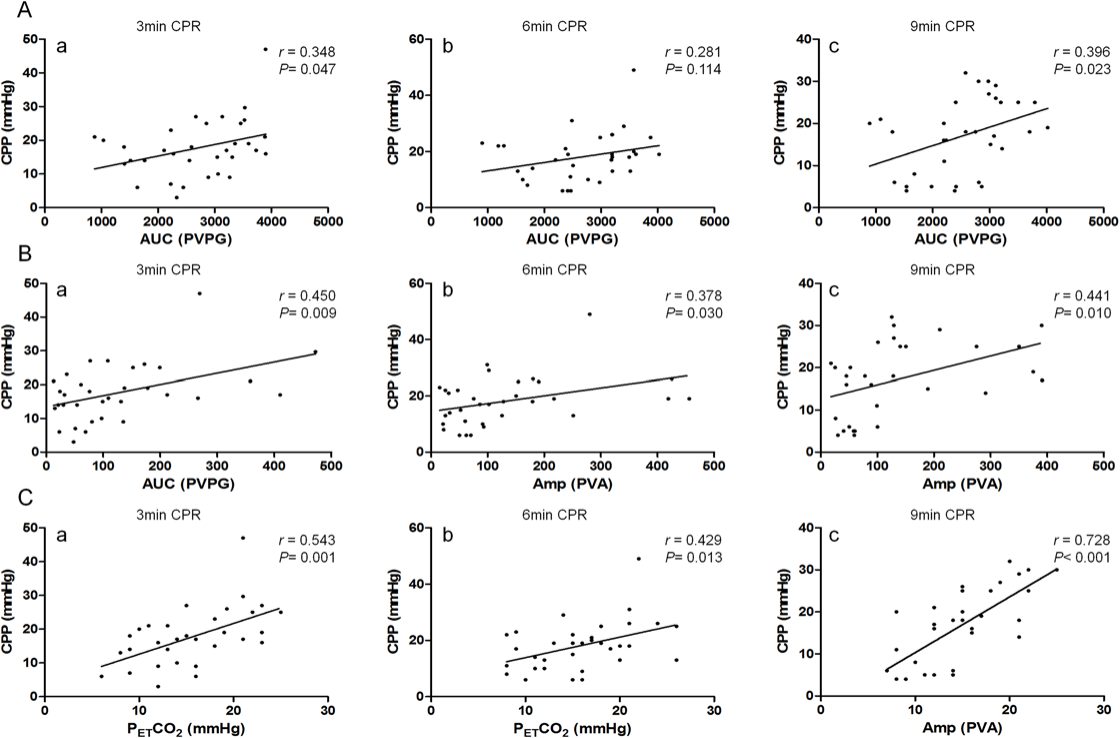
A. The correlation between AUC and CPP during different stages of CPR a. AUC was not correlated with CPP at the 3^rd^ minute of CPR (*r* = 0.348, *P* = 0.047). b. At the 6^th^ minute of CPR, AUC was not correlated with CPP (*r* = 0.281, *P* = 0.114). c. A correlation couldn’t be detected between AUC and CPP at the 9^th^ minute of CPR (*r* = 0.396, *P* = 0.023). B. The correlation between Amp and CPP during different stages of CPR a. Amp was positively correlated with CPP at the 3^rd^ minute of CPR (*r* = 0.450, *P* = 0.009). b. At the 6^th^ minute of CPR, Amp was not correlated with CPP (*r* = 0.378, *P* = 0.030). c. A positive correlation could be detected between Amp and CPP at the 9^th^ minute of CPR (*r* = 0.441, *P* = 0.010). C. The correlation between P_ET_CO_2_ and CPP during different stages of CPR a. P_ET_CO_2_ was positively correlated with CPP at the 3^rd^ minute of CPR (*r* = 0.543, *P* = 0.001). b. At the 6^th^ minute of CPR, P_ET_CO_2_ was not correlated with CPP (*r* = 0.429, *P* = 0.013). c. P_ET_CO_2_ was positively correlated with CPP at the 9^th^ minute of CPR (*r* = 0.728, *P*<0.001).

**Fig 4:**
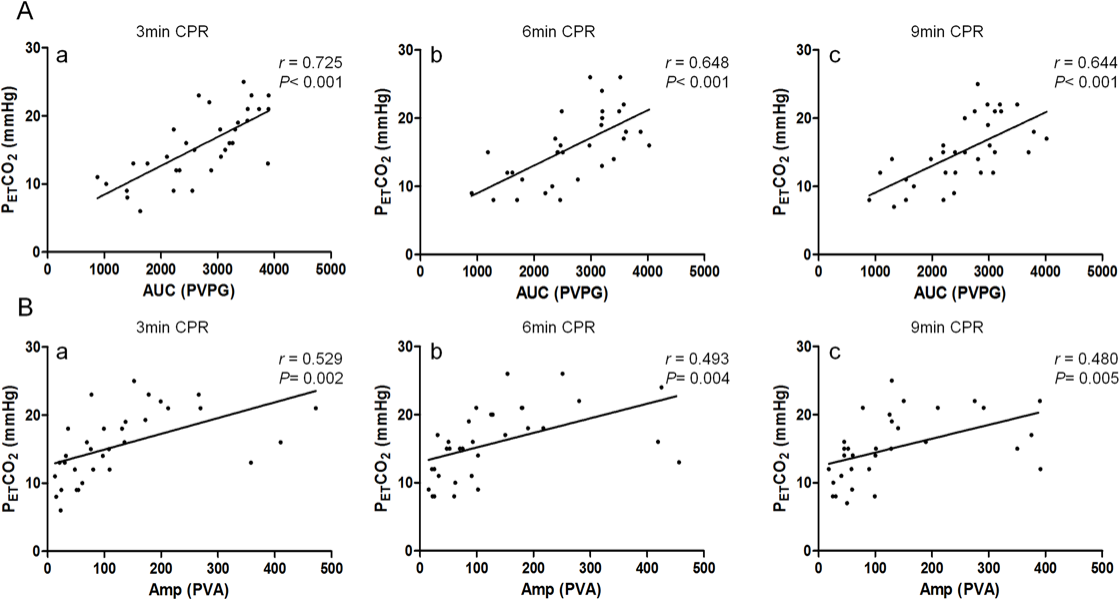
A.The correlation between AUC and P_ET_CO_2_ during different stages of CPR a. AUC was positively correlated with P_ET_CO_2_ at the 3^rd^ minute of CPR (*r* = 0.725, *P*<0.001). b. At the 6^th^ minute of CPR, AUC was positively correlated with P_ET_CO_2_ (*r* = 0.648, *P*<0.001). c. AUC was positively correlated with P_ET_CO_2_ at the 9^th^ minute of CPR (*r* = 0.644, *P*<0.001). B. The correlation between Amp and P_ET_CO_2_ during different stages of CPR a. Amp was positively correlated with P_ET_CO_2_ at the 3^rd^ minute of CPR (*r* = 0.529, *P* = 0.002). b. At the 6^th^ minute of CPR, Amp was positively correlated with P_ET_CO_2_ (*r* = 0.493, *P* = 0.004). c. At the 9^th^ minute of CPR, Amp was positively correlated with P_ET_CO_2_ (*r* = 0.480, *P* = 0.005).

AUC was not correlated with CPP in this study (*r* = 0.348, 0.281 and 0.396, *P* = 0.047, 0.114, and 0.023 at the 3^rd^, 6^th^ and 9^th^ minute respectively) (Fig 3A). Amp was positively correlated with CPP at the 3^rd^ minute (*r* = 0.450, *P* = 0.009) but no correlation was detected at the 6^th^ minute (*r* = 0.378, *P* = 0.030) and 9^th^ minute (*r* = 0.441, *P* = 0.010) (Fig 3B). CPP was positively correlated with P_ET_CO_2_ at the 3^th^ minute (*r* = 0.543, *P* = 0.001) and 9^th^ minute (*r* = 0.728, *P*<0.001), but no correlation was detected at the 6^rd^ minute (*r* = 0.429, *P* = 0.013) (Fig 3C). There was positive correlation between AUC and P_ET_CO_2_ (*r* = 0.725, 0.648, and 0.644 at the 3^rd^, 6^th^ and 9^th^ minute respectively, *P*<0.001) (Fig 4A), the same between Amp and P_ET_CO_2_ (*r* = 0.529, 0.493 and 0.480, *P* = 0.002, 0.004, and 0.005 at the 3^rd^, 6^th^ and 9^th^ minute respectively) (Fig 4B).

During resuscitation, the *F*_*CPR*_ seen on POP was compared to the frequency of mechanical CPR (105 compressions per min) in both groups regardless of compression depth. There was no statistical difference between the *F*_*CPR*_ and the frequency of mechanical CPR at the 3^th^ minute of CPR (104±5, *P* = 0.209). The *F*_*CPR*_ was lower than the frequency of mechanical CPR at the 6^th^ (103±3, *P*<0.001) and the 9^th^ (103±3, *P*<0.001) minute of CPR. The POP reflected the heart beat or effective chest compressions beat-by-beat, as shown in Fig 2.

## Discussion

High quality chest compressions are the cornerstone of cardiopulmonary resuscitation. When rescuers compress to a depth of <38 mm, survival-to-discharge rates after out-of-hospital arrest are reduced by 30%[13]. Similarly, when rescuers compress too slowly, ROSC after in-hospital cardiac arrest falls from 72% to 42%[14]. But the actual quality of chest compressions including compression depth and frequency during both outside-hospital and in-hospital CPR is frequently sub-optimal. The 2010 AHA Guidelines for CPR strongly emphasized that delivering high quality chest compressions is essential, which includes pushing to a depth of at least 5 cm at a rate of at least 100 compressions per minute, allowing full chest recoil, and minimizing interruptions in chest compressions[1]. In order to improve the quality of CPR further, a consensus statement “CPR Quality: Improving Cardiac Resuscitation Outcomes Both Inside and Outside the Hospital” from the American Heart Association was released again in June 2013[15]. Real-time CPR monitoring and feedback to rescuers is critical.

As far as we know, there are two types of CPR quality monitoring systems: physiological monitors (for monitoring hemodynamic effects) and CPR performance metrics (for monitoring the work of the rescuers), both of which can provide real-time feedback. CPP, a surrogate for myocardial perfusion, defined as the aortic diastolic pressure minus the right atrial diastolic pressure, is an example of one the first types. Higher CPP is associated with and predictive of ROSC during CPR both in cardiac arrest patients and in animal models[16–19]. Although CPP is the gold standard for monitoring CPR quality, the use of CPP is still limited because measuring both the aortic diastolic pressure and the right atrial diastolic pressure are invasive and time-consuming, and rarely feasible in resuscitation situations. Pulse oximetry, which reflects the peripheral tissue perfusion status, is similar in appearance to an arterial blood pressure waveform, but what makes it different is that it is noninvasive, inexpensive and nearly ubiquitous in hospitals. As a result, the extraction of circulatory information from POP has been a popular subject of contemporary research.

Anecdotal clinical evidence told us that the higher the quality of CPR was performed, the better the perfusion of peripheral tissue was, and, as a result, the better the POP waveform appeared[20]. In this study, the CPP, AUC and Amp of POP dropped to zero in VF and increased when CPR was performed, even though it was still lower than the initial stage. It was worth mentioning that all these parameters were statistically higher in the high-quality CPR group than in the low quality CPR group. Also, the relationship of AUC and Amp with CPP was analyzed and not exactly correlation between the two parameters was detected, which because CPP monitoring the coronary perfusion, while AUC and Amp of POP the peripheral one. All these results revealed the fact that not only CPP, but also AUC and Amp might be used as a potential marker of high quality CPR.

The 2010 AHA Guidelines for CPR strongly recommended to monitor the quality of the CPR and identify ROSC, and indeed P_ET_CO_2_ has more clinical operability than CPP. Although the optimal target for P_ET_CO_2_ during CPR had not been established[21], a value of less than 10mmHg had been associated with a failure to achieve ROSC and might indicate that the quality of chest compressions should be improved[22]. Studies also showed that P_ET_CO_2_ directly correlated with cardiac output in the cardiac arrest patient[23]. Here, we also found that P_ET_CO_2_ positively correlated with CPP which was consistent with what had been previously reported[24]. In this study, the AUC and Amp of POP were positively correlated with P_ET_CO_2_, which also suggested that the AUC and Amp were a potential clinical way to monitor the quality of CPR. In clinical practice, capnography also had some limitations: it needed advanced airway and calibration which were time-consuming, and it could be easily affected by airway secreta and drugs such as vasoactive agents and sodium bicarbonate administration[25, 26]. Parameters of POP could be used as additive information to P_ET_CO_2_ in the quality monitoring of CPR. And these parameters could be used earlier than P_ET_CO_2_ to monitor the quality of CPR before the construction of the advanced airway.

The impact of chest compressions might influence the stability of the Amp of POP but not the AUC, which reflected the perfusion of peripheral tissue of the whole CPR process instead of the peak point of the process. This result suggested that the AUC might be a more stable parameter than the Amp of POP even though both of them could be used to monitor the quality of CPR non-invasively.

As for the second CPR metric (performance), there were some devices available, such as Q-CPR (HeartStart MRx, Philips). These kinds of devices provided real-time feedback of sternal force and displacement, and an audible signal was produced as an aid to maintaining the desired compression rate and duration, and, as a result, were capable of measuring, recording, and displaying multiple physical quantities during CPR[27]. However, these devices did not correct for any compression of an underlying mattress which might lead to significant under-compression of the chest during CPR[28, 29]. As a system for monitoring the pulsatile volume of blood in tissue instead of measuring the physical movement mechanically, the POP could reflect the quality of CPR without worrying about any such direct effects of the location (e.g. mattress thickness).

Besides the depth of chest compressions, minimizing interruptions and maintaining a high compression rate (100-120bpm) were two other recommended components of high-quality CPR[30]. In this study, pauses in effective compressions showed-up as the absence of waveforms. And the compression rate calculated according to the POP was very similar to the rate of the mechanical compression device. The rate seen on POP decreased over time during CPR, which showed that not all effective peripheral hemoperfusion could be made by chest compression when CPR lasting. So the rate of waveforms could reflect the rate of effective compressions. Therefore, the POP could also be used to monitor the frequency and interruption of CPR in real time, and as a result, the chest compression fraction of CPR could be calculated.

Even though the present findings about POP were interesting, there were also several limitations. First, pulse oximetry itself had some environmental limitations. Strong lights in the room, nail polish, movement of the testing part and other factors could affect the accuracy of the test. Also, the calculation of AUC and Amp of POP was not real-time and needed to be analyzed via separate computer software. However, real-time monitoring software of POP is under development. Third, the perfusion of the peripheral tissue which could affect the parameters of POP could be affected by the neural and humoral factors. However, when cardiac arrest happened, all the regulatory mechanisms were impaired and weakened, and as a result, the perfusion of the peripheral tissue of all patients might go to a trend of homogeneity and could be only affected by the chest compression.

## Conclusions

In summary, the AUC and Amp of POP increased during CPR in cardiac arrested animal models and were higher in the high-quality CPR group than in the low-quality group. They correlated well with existing CPR quality monitoring parameters such as CPP and P_ET_CO_2_, and the AUC was much more stable than the Amp. The frequency of POP reflected the performed chest compressions. AUC and Amp of POP might be used as potential noninvasive quality monitoring parameters for CPR.

## Supporting Information

S1 Minimal Data Set This file includes all the data in the Manuscript. Line 29, ID 27, marked red, was removed because of the extreme values.
(XLS)
